# Synthesis and Degradation of Schiff Bases Containing Heterocyclic Pharmacophore

**DOI:** 10.3390/ijms16011711

**Published:** 2015-01-13

**Authors:** Ionuţ Ledeţi, Anda Alexa, Vasile Bercean, Gabriela Vlase, Titus Vlase, Lenuţa-Maria Şuta, Adriana Fuliaş

**Affiliations:** 1 Faculty of Pharmacy, Victor Babeş University of Medicine and Pharmacy, 2 Eftimie Murgu, Timisoara 300041, Romania; E-Mails: ionut.ledeti@umft.ro (I.L.); suta.lenuta@umft.ro (L.-M.S.); 2 Faculty of Medicine, Victor Babeş University of Medicine and Pharmacy, 2 Eftimie Murgu, Timisoara 300041, Romania; E-Mail: anda_creanga@yahoo.com; 3 Faculty of Industrial Chemistry and Environmental Engineering, Politehnica University of Timişoara, 6 Carol Telbisz, Timisoara 300001, Romania; E-Mail: vbercean@gmail.com; 4 Research Centre for Thermal Analysis in Environmental Problems, West University of Timişoara, Timisoara 300115, Romania; E-Mails: gvlase@cbg.uvt.ro (G.V.); tvlase@cbg.uvt.ro (T.V.)

**Keywords:** Schiff base, synthesis, triazole, thermal behavior, kinetic study

## Abstract

This paper reports on the synthesis and characterization of two Schiff bases bearing 1,2,4-triazolic moieties, namely 4*H*-4-(2-hydroxy-benzylidene-amino)-5-benzyl-3-mercapto-1,2,4-triazole and 4*H*-4-(4-nitro-benzylidene-amino)-5-benzyl-3-mercapto-1,2,4-triazole using thin layer chromatography, melting interval, elemental analysis, spectroscopy and thermal stability studies.

## 1. Introduction

Various derivatives of 1,2,4-triazolic nucleus, such as Schiff bases were investigated in the last decades, for the great variety of medical applications such as antimicrobial [1], hypoglycemic [2], anti-inflammatory [3], anticancer [4], fungicidal [5], anticonvulsant [6] and antiproliferative [7] activities. Schiff bases derived from 1,2,4-triazole are also studied for different technical purposes, such as corrosion inhibition for mild steel in acidic medium [8] or use as magnetic materials and photo-chemically driven molecular devices [9]. In some previous studies, we reported the synthesis of functionalized 1,2,4-triazoles [10,11,12].

Some synthetic pathways were reported for obtaining Schiff bases, such as using catalytic quantities of bivalent transition metal nitrates (M(NO_3_)_2_•*x*H_2_O, M = Cu, Ni, Mn) in organic solvents (MeOH, EtOH, DMF, CH_3_CN) at room temperature [13], the use of acetic acid [14], ethanol without any catalysts [15,16] or in the presence of few drops of concentrated sulfuric acid [17], hydrochloric acid [18], piperidine [19] or an excess amount of concentrated sulfuric acid [20].

Recent literature data mentions the synthesis of 4*H*-4-amino-5-benzyl-3-mercapto-1,2,4-triazole as a potential antibacterial and antifungal compound [21], by the reaction of potassium 3-phenylacetyl-dithiocarbazate with hydrazine hydrate under reflux.

In this study, we described the synthesis and characterization of two Schiff bases (SBs) that contain a functionalized 1,2,4-heterocyclic moiety,* i.e.*, the compounds (SB1) and (SB2), followed by the analysis of their thermal behavior under heating in non-isothermal conditions and completed by the evaluation of the kinetic parameters which characterize their decomposition. In order to realize this study following some previous papers from our research group [22,23,24,25,26,27,28], we used the DTG (derivative thermogravimetry) data obtained at five heating rates in air. We analyzed the decomposition process of Schiff bases occurring in 150–350 °C temperature domain, at heating rates β = 5, 7, 10, 12 and 15 °C·min^−1^. The kinetic triplet was determined according to Kissinger-Akahira-Sunose, Flynn-Wall-Ozawa, Friedman and NPK (non-parametric kinetics) methods. The importance of evaluating the thermal stability and kinetic of decomposition in solid state is essential, especially when the analysis is carried out for compounds having similar structures; in this case, correlations between structure-properties lead to preliminary information over stabilizing/destabilizing moieties from organic compounds.

## 2. Results and Discussion

### 2.1. Synthesis of Schiff Bases

**Scheme 1:**
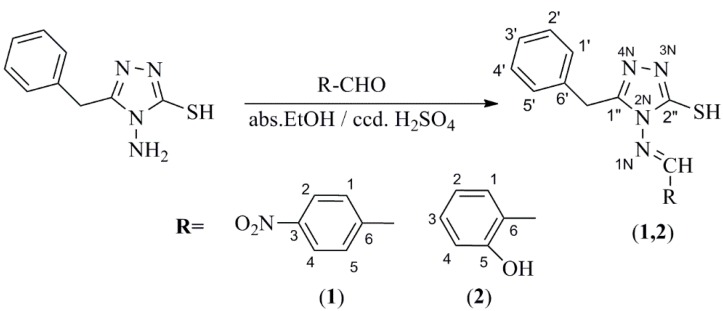
Synthesis procedure for obtaining Schiff bases (SB1) and (SB2).

Substituted aromatic aldehyde (1 mmol) was dissolved in absolute EtOH (20 mL), then 4*H*-4-amino-3-mercapto-5-benzyl-1,2,4-triazole (1 mmol) was added and stirred at room temperature for 15 min. The reaction mixture was cooled at 0–5 °C in an ice bath, then concentrated H_2_SO_4_ (0.75 mL) was added. The suspension was stirred for 4 h, then the solid Schiff bases were separated by filtration (Scheme 1).

### 2.2. Characterization of Compounds

The two Schiff bases were prepared according to literature [10], leading to 4*H*-4-(4-nitro-benzylidene-amino)-5-benzyl-3-mercapto-1,2,4-triazole (SB1) and 4*H*-4-(2-hydroxy-benzylidene-amino)-5-benzyl-3-mercapto-1,2,4-triazole (SB2). Both compounds were obtained as highly-pure compounds using as catalyst a high excess of concentrated sulfuric acid (15:1 mol/mol acid:triazole). Both derivatives were characterized by melting point (m.p.), thin-layer chromatography (TLC) and Fourier-transform infrared (FTIR) spectroscopy (Perkin-Elmer Spectrum 100 FT-IR Spectrometer with Universal Attenuated Total Reflectance (UATR) device).

#### 2.2.1. Physico-Chemical Analysis of Schiff Bases

Characterization of Compound (SB1): 4*H*-4-(4-nitro-benzylidene-amino)-5-benzyl-3-mercapto-1,2,4-triazole.

Yellow powder (yield η = 60%), m.p. = 235–238 °C.

FTIR (KBr, cm^−1^): *ν*^asoc^ (absorbed water) (OH) = 3689–3252 (broad, 3496 (i)), *ν*^car^ (H) = 3093 (m), 3053 (m), *ν*^as^ (CH_2_) = 2942 (i), *ν*^s^ (CH_2_) = 2902 (m), 2761 (m), ν_sk, ar_ = 1596 (m), 1575 (m), ν^as^ (NO_2_) = 1514 (i), ν^s^ (NO_2_) = 1343 (i), γ_sk, ar_ = 826 (m), 724 (m).

UV-VIS: λ_max_ (nm, THF): (ε, L·mol^−1^·cm^−1^): 360 (5170).

Elemental analysis (Calcd./Found) for C_16_H_13_N_5_O_2_S, M = 33,937 (g/mol): C = 56.63%, H = 3.86%, N = 20.64%; Found: C = 56.25%, H = 3,59%, N = 19.98%.

^1^H-RMN δ_H_ (DMSO-*d*_6_, 600.13 MHz): 3.4 (CH_2_); 8.33 (2-H, 4-H); 7.21 (3'-H); 7.14-7.29 (1'-H, 2'-H, 4'-H, 5'-H); 8.09 (1-H, 5-H), 10.41 (N=CH).

^13^C-RMN δ_C_ (DMSO-*d*_6_, 150.92 MHz): 30.5 (CH_2_); 124.1 (2-C, 4-C); 126.9 (3'-*C*); 128.5, 128.8 (1'-C, 2'-C, 4'-C, 5'-C); 129.5 (1-C, 5-C), 134.9 (6'-C), 138.1 (6-C); 150.7 (1''-C); 158.4 (N = CH); 161.5 (2''-C).

^15^N-RMN δ_C_ (DMSO-*d*_6_, 60.82 MHz): 201.9 (3-N); 208.9 (2-N); 282.4 (4-N); 311.4 (1-N); 369.7 (NO_2_).

Characterization of Compound (SB2): 4*H*-4-(2-hydroxy-benzylidene-amino)-5-benzyl-3-mercapto-1,2,4-triazole.

White powder (yield η = 65%), m.p. = 193–198 °C.

FTIR (KBr, cm^−1^): *ν*^asoc^ (OH) = 3353i; *ν*^car^ (H) = 3105, 3066 (m), 2938 (m); *ν*^as^ (CH_2_) = 2855, 1621; ν_sk, ar_ = 1603 (m), 1586 (m), 1453 (m); γ_sk, ar_ = 760 (m), 714 (m).

Elemental analysis (Calcd./Found) for C_16_H_14_N_4_OS, M = 31,037 (g/mol): C = 61.92%, H = 4.55%, N = 18.05%; Found: C = 61.68%, H = 4,31%, N = 17.73%.

^1^H-RMN δ_H_ (DMSO-*d*_6_, 600.13 MHz): 4.14 (CH_2_); 6.93 (2-H); 6.99 (4-H); 7.20 (3'-H); 7.84 (1-H); 7.27 (1'-H, 2'-H, 4'-H, 5'-H); 7.40 (3-H); 10.23 (N=CH).

^13^C-RMN δ_C_ (DMSO-*d*_6_, 150.92 MHz): 30.7 (CH_2_); 116.6 (4-C); 118.3 (5-C); 119.6 (2-C); 126.8 (3'-C); 127.4 (1-C); 128.4, 128.8 (1'-C, 2'-C, 4'-C, 5'-C); 134.1 (3-C); 135.1 (6'-C); 150.3 (1''-C); 158.4 (6-C); 160.1 (N=CH); 161.5 (2''-C)

^15^N-RMN δ_C_ (DMSO-*d*_6_, 60.82 MHz): 200.0 (3-N); 210.3 (2-N); 268.3 (4-N); 295.4 (1-N).

#### 2.2.2. Spectroscopic Analysis

The recorded NMR signals for both Schiff bases confirm their structure. In order to confirm the formation of condensation products between starting materials (namely the amino-triazole and the corresponding aldehyde), ^1^H-NMR, ^13^C-NMR and ^15^N-NMR spectra were drawn up. The formation of the Schiff base SB1 is confirmed by NMR spectroscopy, when a signal corresponding to the bonded atoms in the N=CH moiety are observed for nitrogen, proton and carbon. The presence of two phenyl groups in the structure is confirmed by the signals of the aromatic protons in the spectral range 7.14–7.29 (corresponding to five protons 1'-H to 5'-H), and 8.33 ppm and 8.09 ppm (corresponding to two pairs of protons, 1-H, 5-H and 2-H, 4-H). This also confirms the substitution in *para* position from the azomethynic moiety. The presence of nitrogen atoms in the structure is another confirmation of the synthesis of Schiff base, ^15^N-NMR spectroscopy revealing the presence of five type of nitrogens: three endocyclic from 1,2,4-triazol nucleus, one from the azomethyn bond and the one from the nitro group. A similar discussion can be carried out for the SB2 derivative. In this case too, the presence of two aromatic phenyl groups are confirmed by signals from proton and carbon spectra, the azomethynic bond is confirmed, as well the substitution of the aldehyde moiety in the *ortho*-position. Also, ^15^N-NMR spectroscopy confirms the presence of three endocyclic nitrogens from the heterocyclic moiety, as well as the presence of the azomethynic nitrogen.

**Figure 1:**
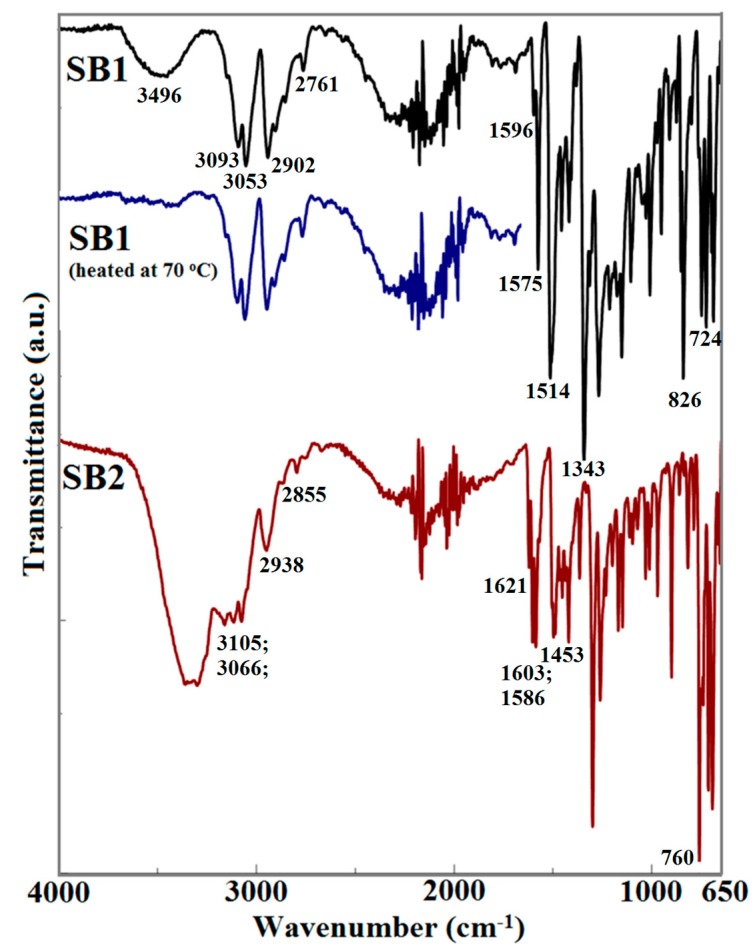
The FTIR spectra of Schiff bases: SB1, SB1 heated at 70 °C and SB2.

The FTIR spectra of the two Schiff bases are presented in Figure 1. Analysis of the spectra corresponding to Schiff base SB1 reveals the appearance of several characteristic bands. A broad band in the spectral range 3689–3252 cm^−1^, with a maximum at 3496 cm^−1^ cannot be associated with the presence of a structural moiety from the compound. This band is associated with the adsorbed water molecules. The presence of water molecules was demonstrated both in the analysis of the TG (thermogravimetric) curve, and was confirmed by FTIR analysis for the sample of SB1 derivative after heating at 70 °C. In this case, the broad band disappeared from the spectrum (Figure 1). At higher wavenumbers, the bands associated with stretching vibrations of the aromatic C–H bond at 3093 and 3053 cm^−1^, as well the symmetric and asymmetric vibrations corresponding to the CH_2_ group at 2902 and 2761 cm^−1^ can be identified in the spectrum. Other important bands can be associated with the vibrations of the aromatic skeleton (1596 and 1575 cm^−1^), while for the nitro group, two intense bands at 1514 and 1343 cm^−1^ can be noticed in the spectrum. The out of plane bending for the aromatic skeleton can be seen at 826 and 724 cm^−1^.

The analysis of the FTIR spectra of the SB2 derivative lead to the conclusion that the Schiff base derivative was obtained. The presence of the –OH moiety in the structure of the Schiff base is indicated by the presence of a broad and intense band in 3600–2800 cm^−1^ spectral region, corresponding to stretching vibrations of H-bond associated hydroxyl groups. The stretching vibrations of the aromatic C–H bond are observed at 3105, 3066 and 2938 cm^−1^ as medium-weak bands, as well as the symmetric and asymmetric vibrations corresponding to the CH_2_ group at 2855 and 1621 cm^−1^. Other important bands can be associated with the vibrations of the aromatic skeleton (1603, 1586 and 1453 cm^−1^), while the out of plane bending for the aromatic skeleton can be seen at 760 and 714 cm^−1^.

#### 2.2.3. Thermal Analysis

**Figure 2:**
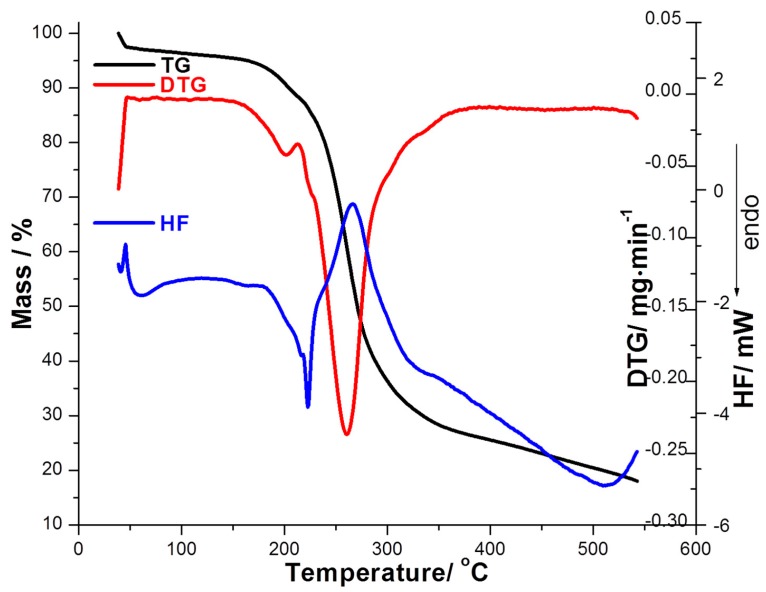
The thermoanalytical curves (TG/DTG/HF) recorded with β = 15 °C/min for SB1 derivative. TG: thermogravimetric; DTG: derivative thermogravimetry; HF: heat flow.

The thermoanalytical curves of Schiff bases Equations (1) and (2) were obtained during heating at β = 15 °C·min^−1^ in air atmosphere and exhibit multistadial decomposition route. Schiff base (SB1) loses adsorbed surface water (Δ*m* = 4.84%) up to 62 °C, which is accompanied by an endothermic peak on *HF* (heat flow) curve with *HF*_max_ at 58 °C. This compound is then thermally stable up to 160 °C, when a decomposition process begins and continues up to 310 °C, with a mass loss Δ*m* = 61.31%. In this temperature range (160–310 °C), one can notice two maximums on the DTG curve (at 200.2 and 261.1 °C). The analysis of the HF curve reveals an endothermic effect (*HF*_max_ = 224 °C) which is clearly associated with the melting and decomposition of the Schiff base, followed by an exothermic effect with *HF*_max_ = 265 °C which is due to the destruction of the molecular structure of the compound. This process of decomposition is continued up to 550 °C, but no constant mass is obtained (Figure 2).

**Figure 3:**
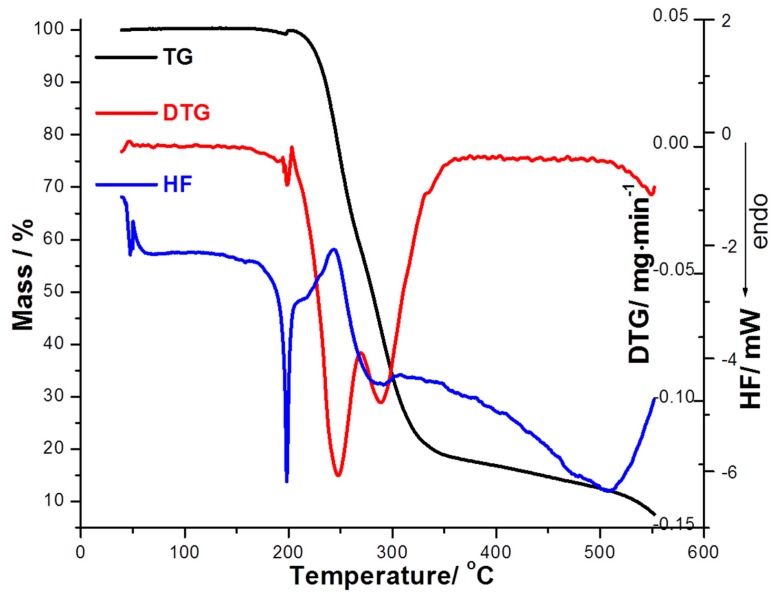
The thermoanalytical curves (TG/DTG/HF) recorded with β = 15 °C/min for SB2 derivative.

For Schiff base (SB2), the analysis of thermoanalytical curves (Figure 3) reveals a better thermal stability (up to 204 °C). Only the HF curve reveals an endothermic effect at 197.7 °C which is due to the melting and is in good agreement with the melting observed on the Boethius apparatus. TG curve indicates that a two steps degradation of this compound occurs, in 204–273 °C (with a mass loss Δ*m* = 44.44% and with a *DTG*_max_ = 247 °C), and in 273–357 °C (with a mass loss Δ*m* = 37.07% and with a *DTG*_max_ = 288 °C) temperature ranges, respectively. The HF curve indicates in these temperature ranges two maximums, at 244 and 286 °C which can be associated with thermooxidation processes of molecule destruction.

In order to evaluate the substituent effect (4-nitro-benzylidene-amino* vs.* 2-hydroxy-benzylidene-amino) grafted to the triazolic nucleus, a kinetic analysis was performed.

#### 2.2.4. Kinetic Study

The kinetic analysis was made using the TG data in air for the decomposition of SB1 and SB2 samples at five heating rates: β = 5, 7, 10, 12 and 15 °C·min^−1^. To perform the kinetic analysis of the TG experimental data, we used three isoconversional methods, a differential one (Friedman) and two integral ones (Flynn-Wall-Ozawa and Kissinger-Akahira-Sunose methods), respectively a fourth method elaborated by Sempere* et al.* [29,30] and modified and developed by Vlase* et al.* [31,32,33], the non-parametric kinetics method (NPK), in order to obtain realistic kinetic parameters.

It can be considered that for an elementary reaction, the expression of the reaction rate can be written as the product of two functions, one dependent on temperature (T) and one dependent on the conversion degree α:

dα/d*t* = *k*(*T*)·*f*(α) (1)

where *t* is time, α is the conversion degree, *T* temperature, *f*(α) the differential conversion function, *k*(*T*) the rate constant. The most verified and widely used equation to determine the constant rate dependence of temperature is the Arrhenius equation:

*k*(*T*) = *A*·exp[−*E_a_*/(*R*·*T*)] (2)

where *A* is the pre-exponential factor; *E_a_* is the activation energy and *R* is the gas constant.

In non-isothermal experiments, it is used a linear temperature program, in which the heating rate is constant:

β = d*T*/d*t* = constant (3)

Equation (3) allows the change in reaction rate equation (Equation (1)) of the variable time with the variable temperature, obtaining the relation:

dα/d*T* = *A*/β·exp[−*E_a_*/(*R*·*T*)]·*f*(α) (4)

##### Flynn-Wall-Ozawa Method (FWO)

Through the integration of Equation (4) [34,35] and using the Doyle approximation [36], FWO relation was obtained as follows:

ln β = ln [*A·E·R*^−1^·g^−1^(α)] − 5.331 − 1.052·*E_a_·R*^−1^·*T*^−1^ (5)

where g(α) is the integral conversion function.

**Figure 4:**
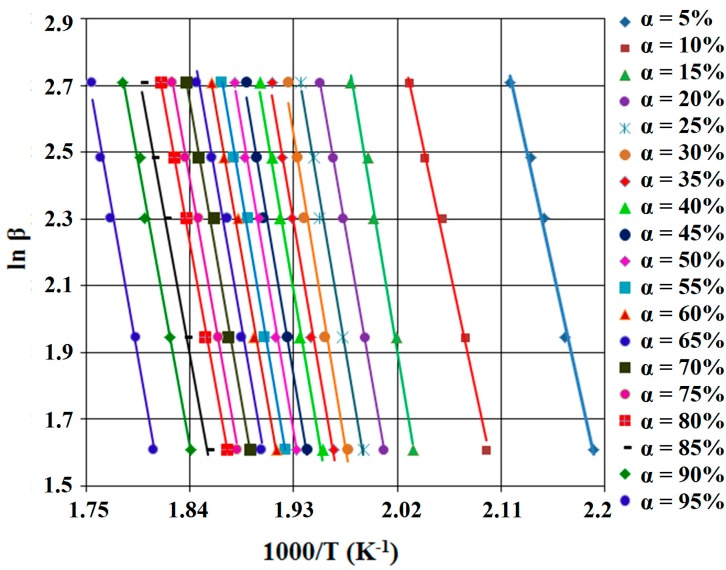
FWO (Flynn-Wall-Ozawa) diagram, for SB1 derivative.

**Figure 5:**
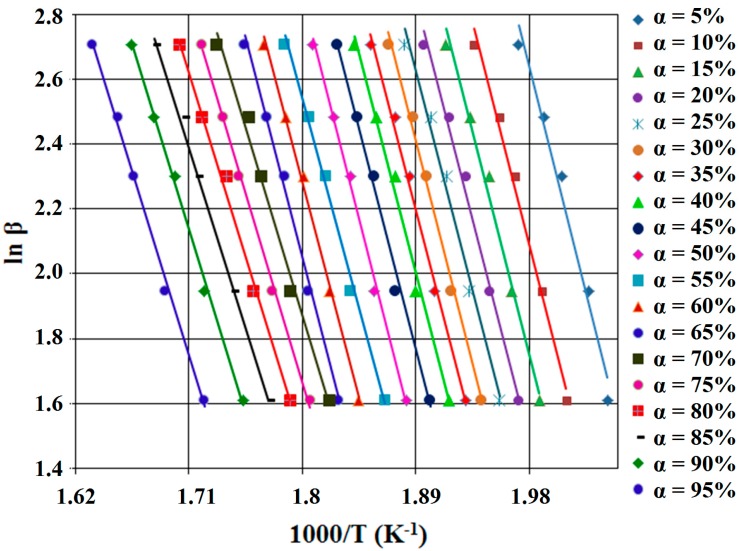
FWO diagram, for SB2 derivative.

According to this equation, for constant value of α of each different β, the value of activation energy (*E_a_*) could be calculated using the linearity between ln β and reciprocal of temperature (T^−1^). Therefore, the calculated activation energy values (*E_a_*) can be obtained through Equation (5) using the slope value of straight lines, nearly parallel, for all the conversion degree, which are presented in Figure 4 and Figure 5.

All resulted values of the activation energy corresponding to the new synthetized compounds (SB1 and SB2) are summarized in Table 1.

**Table 1:** The obtained values for activation energies by the three isoconversional methods for the new Schiff bases.

*E_a_* (kJ/mol)		Conversion Degree (α)																			$\bar{E_{a}}$ (kJ/mol)
		0.05	0.1	0.15	0.2	0.25	0.3	0.35	0.4	0.45	0.5	0.55	0.6	0.65	0.7	0.75	0.8	0.85	0.9	0.95	
**SB1**	**FR**	116.8	122.9	149.4	144.4	135.7	136.6	137.8	139.4	142.2	140.4	136.2	141.5	141.1	140.1	140.9	136.8	131.2	134.4	138.0	137.2 ± 1.7
	**KAS**	122.1	126.4	162.5	155.9	159.3	165.1	160.1	160.7	160.0	160.9	158.8	154.7	154.6	156.8	152.9	151.4	149.6	147.4	152.9	153.3 ± 2.6
	**FWO**	123.4	127.8	162.3	156.1	159.5	165.1	160.3	160.9	160.4	161.3	159.3	155.4	155.4	157.5	153.9	152.5	150.9	148.9	154.2	153.9 ± 2.5
**SB2**	**FR**	114.7	120.8	121.8	118.6	115.3	117.1	118.9	119.5	120.2	118.2	118.1	123.3	126.9	106.7	106.9	106.5	104.2	104.3	96.0	114.6 ± 1.9
	**KAS**	119.8	118.3	117.3	116.5	116.8	118.7	116.3	116.8	117.6	117.2	110.1	114.3	116.2	97.6	97.9	95.4	93.7	95.4	95.2	110.1 ± 2.3
	**FWO**	121.7	120.5	119.6	118.9	119.3	121.1	118.9	119.5	120.3	119.9	113.3	117.4	119.3	101.7	102.1	99.73	98.19	99.91	99.92	113.2 ± 2.1

FR: Friedman Method; KAS: Kissinger-Akahira-Sunose Method; FWO: Flynn-Wall-Ozawa Method.

##### Friedman Method (FR)

This method is a differential isoconversional one and it is based on the Equation (4), by applying logarithm function, leading to Friedman relation [37]:

ln (β·dα/d*T*) = ln [*A*·*f*(α)] − *E_a_*/(*R*·*T*) (6)

**Figure 6:**
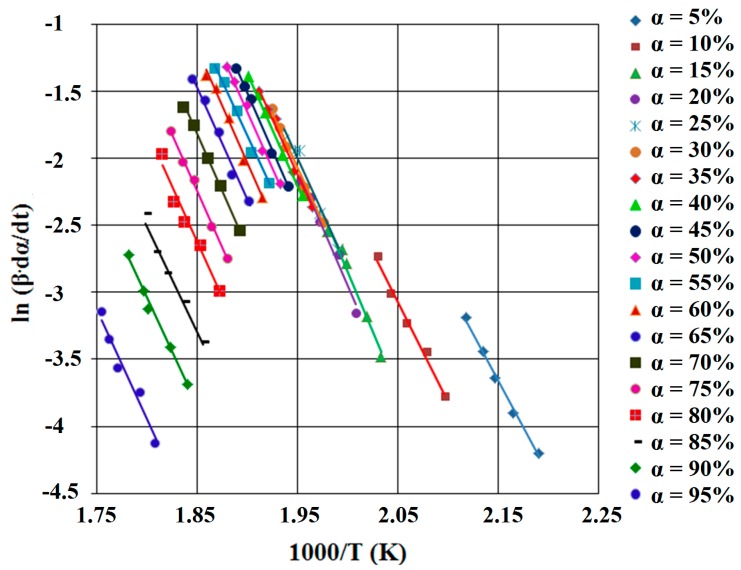
FR (Friedman) diagram, for SB1 derivative.

**Figure 7:**
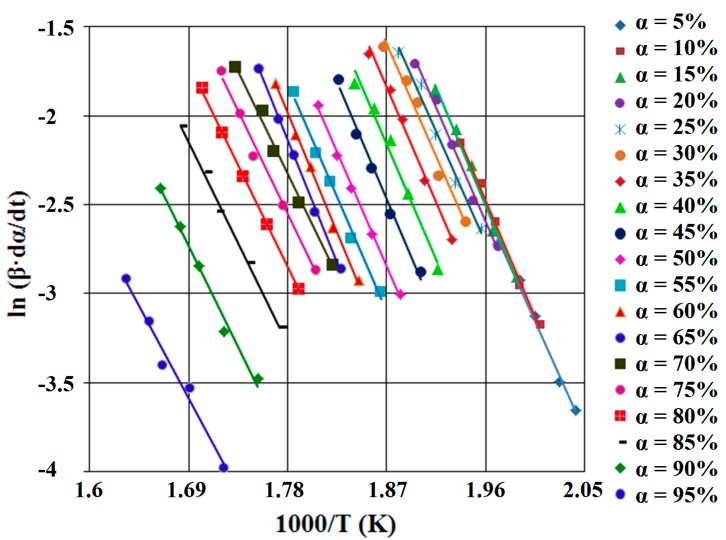
FR diagram, for SB2 derivative.

For α = constant and using the five heating rates, the correlation ln (β·dα/d*T*)* vs.* (1/*T*) is linear. From the slopes of these straight lines which form the Friedman diagram (see Figure 6 and Figure 7), the values of the activation energy (*E_a_*) for the two compounds are obtained (Table 1).

##### Kissinger-Akahira-Sunose Method (KAS)

The third isoconversional method, Kissinger-Akahira-Sunose [38,39], is an integral one which used the integral form of Equation (4) and the Murray and White approximation [40]

ln (β/*T*^2^) = ln [*A*·*R*/(*E_a_*·*g*(α))] − *E_a_*/(*R*·*T*) (7)

**Figure 8:**
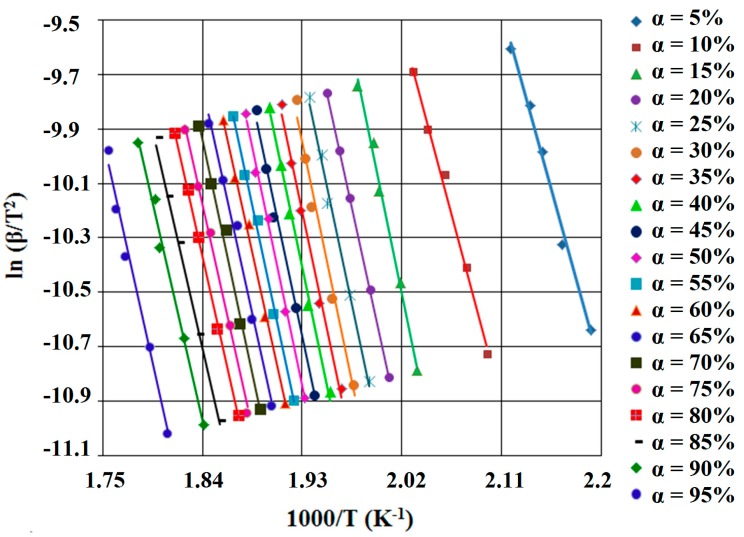
KAS (Kissinger-Akahira-Sunose) diagram, for SB1 derivative.

**Figure 9:**
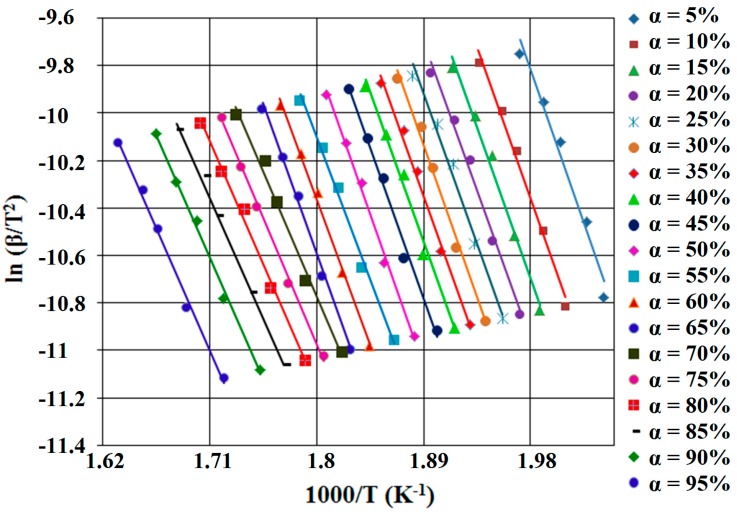
KAS diagram, for SB2 derivative.

Using the thermoanalytical curves recorded at different heating rates β, the plot ln β/*T*^2^* vs.* 1/T lead to straight lines (KAS diagram, see Figure 8 and Figure 9) with a value for *R*^2^ > 0.95. The value of activation energy can be obtained from the slope of the straight lines for each conversion degree, independent of the expression of conversion function. If the value for *R*^2^ < 0.95 for some straight lines or the change of the straight lines’ slope, implicit *E_a_* values function of α, it can be argued that the reaction mechanism changes.

The non-parametric kinetics method (NPK) elaborated by Sempere* et al.* [29,30] and modified and developed by Vlase* et al.* [31,32,33] is based on the assumption that the reaction rate can be expressed as a product of two independent functions, *f*(α): a function of the degree of conversion and *k*(*T*): a temperature dependence, so the validity of Equation (1) is the only assumption made in the development of the NPK method. The reaction rates for several measurements at different β can be written as an *n* × *m* matrix whose rows correspond to constant value of conversion degree and whose columns account for the different but constant values of temperature. This matrix is decomposed into two vectors using Singular Value Decomposition (SVD) algorithm [41].

The results of NPK analysis are systematized in Table 2 and were obtained using a kinetic model suggested by Šesták and Berggren [42]:

*f*(α) = α*^m^*·(1 − α)*^n^* (8)

**Table 2:** The results of kinetic analysis by the NPK method.

	Process	λ (%)	E_a_ (kJ·mol^−1^)	A (s^−1^)	*n*	*m*	Šestak-Berggren Equation	$\bar{E_{a}}$ (kJ·mol^−1^)
SB1	1	66.2	124.0 ± 7.7	1.33 × 10^10^	1/3	-	(1 − α)^1/3^	135.1 ± 10.7
	2	31.4	152.2 ± 2.7	3.19 × 10^15^	1	-	(1 − α)	
	3	2.4	214.5 ± 8.3	9.95 × 10^18^	1	1	(1 − α)·α	
SB2	1	89.9	111.4 ± 7.2	1.66 × 10^10^	1/2	-	(1 − α)^1/2^	109.9 ± 8.2
	2	9.4	104.2 ± 1.1	2.82 × 10^10^	-	1/3	α^1/3^	

**Figure 10:**
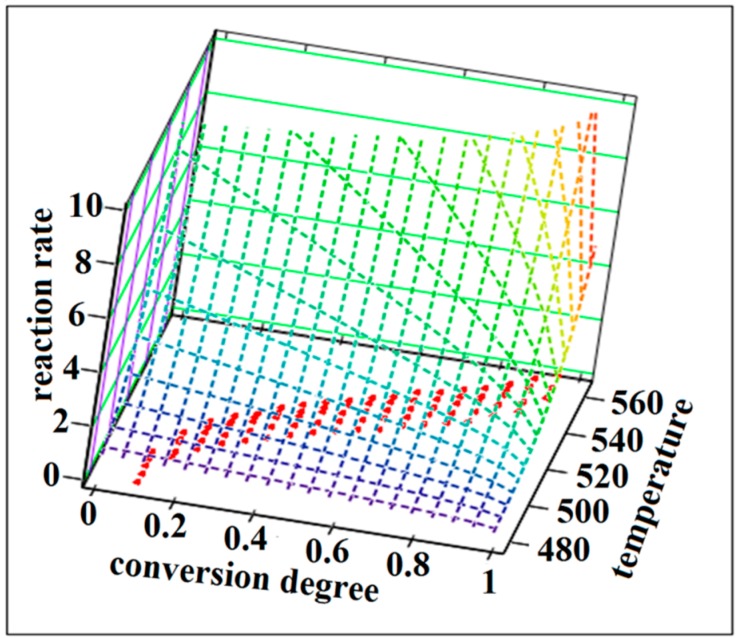
The reaction rate surfaces in 3D space with the coordinates (β·dα/d*T*; α;* T*) for the SB1 compound.

**Figure 11:**
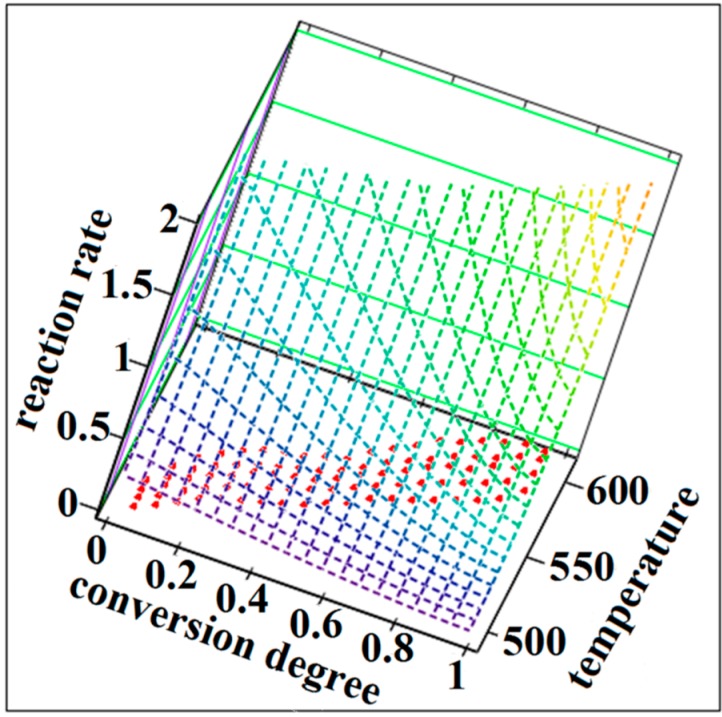
The reaction rate surfaces in 3D space with the coordinates (β·dα/d*T*; α;* T*) for the SB2 compound.

The NPK method is a model-free method because the temperature dependence of the reaction rate and implicit the value of activation energy can be obtained without using approximations or any assumptions about the reaction model. This method involves a lot of experimental points that form a surface in a three dimensional space where the axis are the temperature, the degree of conversion and the rate of change of the degree of conversion (Figure 10 and Figure 11).

The λ parameter from Table 2 represents explained variance and is defined as the contribution of each step to the whole thermodegradation process, so that Σ λ_i_ = 100%. Considering these aspects and analyzing Table 2, it significant differences can be observed in the thermal behavior of these Schiff bases: SB1 compound is thermodegraded into a complex process which implies three steps with different values of activation energy and different significance for kinetic parameters (*n* and *m*); SB2 thermal behavior is characterized by a process with two significant parallel steps. First step is a chemical one with *n* = 1/2 and the second one has a physical nature with *m* = 1/3.

Calculating the mean values of *E_a_* (∑ λ·*E_a_*), the thermal stability of SB1 is higher than SB2, this fact being certainly in connection with the chemical structure of these two Schiff bases.

## 3. Experimental Section

All reagents and solvents were commercial products of Merck (Darmstadt, Germany) and used without further purification. 4*H*-4-amino-3-mercapto-5-benzyl-1,2,4-triazole was prepared and characterized in our laboratory, according to literature [21]. Reduced-pressure evaporation of solvents was realized using a Büchi rotary evaporator R-200 (Büchi, Zurich, Switzerland) equipped with a Büchi heating bath B-490 and coupled to a Rotavac vacuum pump.

Melting points were determined on a Böetius PHMK (Veb Analytik Dresden) instrument and are uncorrected. Thin-layer chromatography (TLC) was performed with silica gel-coated plates 60 F_254_ Merck using benzene:methanol 7:3 (*v*:*v*) and benzene:ethyl acetate 1:1 (*v*:*v*) as eluants. The chromatographic spots were revealed by exposure to iodine vapors and/or UV light irradiation (λ = 254 nm).

FTIR spectra were recorded on a UATR device without previous sample preparation using a Perkin Elmer SPECTRUM 100 device (Perkin-Elmer Applied Biosystems, Foster City, CA, USA). The data was collected in 4000–600 cm^−1^ domain, on an UATR device. Spectra were built up after a number of 24 acquisitions for each spectrum. The UV-VIS spectra were recorded in methanol on a Jasco V-530 spectrophotometer (Jasco, Tokyo, Japan). ^1^H-NMR, ^13^C-NMR and ^15^N-NMR spectra were recorded on a Bruker Avance DRX spectrometer (Bruker BioSpin GmbH, Rheinstetten, Germany) in DMSO-*d*_6_, using TMS as reference, chemical shifts being reported in ppm.

The percentage of C, H and N were obtained by means of elemental analysis using a Vario El Cube apparatus (Elementar Analysensysteme GmbH, Hanau, Germany).

The thermal analysis was carried out on a Perkin-Elmer DIAMOND thermo-balance for obtaining simultaneously the TG, DTG and HF curves, in dynamic air atmosphere (100 mL·min^−1^), using aluminum crucibles. The analyses were carried out under non-isothermal conditions at five heating rates β, namely 5, 7, 10, 12 and 15 °C·min^−1^ from 20 up to 550 °C. For determining the thermal effects, the DTA data (in µV) were converted in HF (Heat Flow) data (mW).

In order to assure the reproducibility of TG study, each analysis was repeated four times. The results were practically identical.

## 4. Conclusions

Two Schiff base derivatives, 4*H*-4-(4-nitro-benzylidene-amino)-5-benzyl-3-mercapto-1,2,4-triazole (SB1) and 4*H*-4-(2-hydroxy-benzylidene-amino)-5-benzyl-3-mercapto-1,2,4-triazole (SB2) were synthesized. The two compounds were obtained with good yields and high purity and were properly characterized by melting interval, thin-layer chromatography, elemental analysis, NMR and FTIR spectroscopy, followed by the characterization of their thermal behavior. The study was completed by the kinetic analysis of solid-state degradation of the two compounds. By comparing the obtained values for activation energies, it was revealed that the SB1 derivative is more stable than SB2 derivative, which can be associated to the substituent effect. In the case of SB1 derivative, the presence of the *para*-nitro moiety, which is an electron-withdrawing group (M mesomeric effect) is more significant that the presence of *ortho*-hydroxy group in the case of SB2 derivative, which is a +M substituent.
